# Capacity To Utilize Raffinose Dictates Pneumococcal Disease Phenotype

**DOI:** 10.1128/mBio.02596-18

**Published:** 2019-01-15

**Authors:** Vikrant Minhas, Richard M. Harvey, Lauren J. McAllister, Torsten Seemann, Anna E. Syme, Sarah L. Baines, James C. Paton, Claudia Trappetti

**Affiliations:** a Research Centre for Infectious Diseases, Department of Molecular and Biomedical Science, University of Adelaide, Adelaide, Australia; b Melbourne Bioinformatics, The University of Melbourne, Melbourne, Australia; c Department of Microbiology and Immunology, The University of Melbourne, Melbourne, Australia; University of Mississippi Medical Center; NYU Lagone Health; Tufts University School of Medicine

**Keywords:** *Streptococcus pneumoniae*, carbohydrate metabolism, otitis media, pneumonia, single nucleotide polymorphisms, virulence

## Abstract

Streptococcus pneumoniae is commonly carried asymptomatically in the human nasopharynx, but it also causes serious and invasive diseases such as pneumonia, bacteremia, and meningitis, as well as less serious but highly prevalent infections such as otitis media. We have previously shown that closely related pneumococci (of the same capsular serotype and multilocus sequence type [ST]) can display distinct pathogenic profiles in mice that correlate with clinical isolation site (e.g., blood versus ear), suggesting stable niche adaptation within a clonal lineage. This has provided an opportunity to identify determinants of disease tropism. Genomic analysis identified 17 and 27 single nucleotide polymorphisms (SNPs) or insertions/deletions in protein coding sequences between blood and ear isolates of serotype 14 ST15 and serotype 3 ST180, respectively. SNPs in raffinose uptake and utilization genes (*rafR* or *rafK*) were detected in both serotypes/lineages. Ear isolates were consistently defective in growth in media containing raffinose as the sole carbon source, as well as in expression of raffinose pathway genes *aga*, *rafG*, and *rafK*, relative to their serotype/ST-matched blood isolates. Similar differences were also seen between serotype 23F ST81 blood and ear isolates. Analysis of *rafR* allelic exchange mutants of the serotype 14 ST15 blood and ear isolates demonstrated that the SNP in *rafR* was entirely responsible for their distinct *in vitro* phenotypes and was also the determinant of differential tropism for the lungs versus ear and brain in a mouse intranasal challenge model. These data suggest that the ability of pneumococci to utilize raffinose determines the nature of disease.

## Abstract (precis)

S. pneumoniae is a component of the commensal nasopharyngeal microflora of humans, but from this reservoir, it can progress to localized or invasive disease with a frequency that translates into massive global morbidity and mortality. However, the factors that govern the switch from commensal to pathogen, as well as those that determine disease tropism, are poorly understood. Here we show that capacity to utilize raffinose can determine the nature of the disease caused by a given pneumococcal strain. Moreover, our findings provide an interesting example of convergent evolution, whereby pneumococci belonging to two unrelated serotypes/lineages exhibit SNPs in separate genes affecting raffinose uptake and utilization that correlate with distinct pathogenic profiles *in vivo*. This further underscores the critical role of differential carbohydrate metabolism in the pathogenesis of localized versus invasive pneumococcal disease.

## INTRODUCTION

Streptococcus pneumoniae (the pneumococcus) is one of the world’s foremost bacterial pathogens, killing 1 to 2 million people each year. In spite of this, it is considered part of the “normal” nasopharyngeal microflora, asymptomatically colonizing up to 65% of individuals; these carriers are the principal reservoirs for transmission of S. pneumoniae in the community (1, 2). In a small proportion of carriers, which nevertheless translates into globally significant numbers, S. pneumoniae invades from its nasopharyngeal reservoir to cause disease: e.g., by aspiration into the lungs to cause pneumonia, by direct or indirect invasion of the blood (bacteremia) or central nervous system (meningitis), or by ascension of the eustachian tube to access the middle ear and cause otitis media (OM) (1, 2). However, the molecular mechanisms whereby pneumococci transition from a commensal lifestyle to cause either localized or invasive disease are poorly understood.

The pneumococcus is a genetically plastic and diverse species, comprising at least 98 capsular serotypes, superimposed on more than 12,000 clonal lineages (sequence types [STs]) recognizable by multilocus sequence typing (3). It has a core genome of roughly 1,500 genes, with the remaining 30% of the genome present as accessory regions (ARs), present in some but not all clonal lineages. Individual S. pneumoniae strains can differ markedly in their virulence phenotypes, including their capacity to colonize the nasopharynx, spread from person to person, or progress to either localized or invasive infections. Capsule switching experiments have shown that both serotype and genetic background (i.e., ST) inﬂuence virulence (4, 5), but strain complexity has complicated attempts to examine whether there is any association between a given clonal lineage or serotype and propensity to cause localized rather than invasive infections.

Previous studies in our laboratory have shown that S. pneumoniae clinical isolates belonging to the same serotype and ST may display distinct virulence phenotypes in mice, in accordance with their original site of isolation in humans (blood versus ear). After intranasal (i.n.) challenge, serotype 3 blood isolates belonging to ST180, ST232, and ST233 did not stably colonize the nasopharynx, but spread to the blood in the majority of mice; none spread to the ear. In contrast, ear isolates colonized the nasopharynx at higher levels than the respective ST-matched blood isolates and also spread to the ear compartment; none caused bacteremia (6). In a separate study, serotype 14 (ST15) blood and ear isolates all exhibited a similar capacity to colonize the nasopharynx, but significant differences were observed between bacterial loads in other host niches. Blood isolates caused pneumonia in most animals, whereas ear isolates were not detected in the lungs of any of the mice 24 h post-intranasal challenge. Conversely, ST15 ear isolates, but not blood isolates, were able to spread to the brain, and in the ear compartment, the bacterial load and proportion of infected mice were significantly greater for mice challenged with ear rather than blood isolates (7). Thus, strains within a clonal lineage appear to be exhibiting stable niche adaptation.

Although members of the same serotype and ST type have very closely related genetic backbones, they are not necessarily identical and may have acquired distinct ARs or other genetic changes, such as single nucleotide polymorphisms (SNPs) or insertions or deletions (indels). In the present study, we have compared the genomes of representative serotype 14 ST15 and serotype 3 ST180 blood and ear isolates to determine whether such differences can account for their distinct virulence phenotypes. We show that SNPs in loci responsible for uptake and utilization of the trisaccharide raffinose are the ultimate determinant of disease progression.

## RESULTS

### Genetic differences between serotype/ST-matched blood and ear isolates.

In the first instance, draft genomes of serotype 14 ST15 strains 4559 (blood isolate) and 947 (ear isolate) were assembled from PacBio and MiSeq data and then compared (see Materials and Methods). The only differences in ARs were the presence of a 35-kb prophage and a 3.2-kb plasmid in 4559 and not 947, but these ARs were not present in other serotype 14 ST15 blood isolates in our collection (results not shown). Seventeen SNPs and indels present within protein coding sequences of 4559 and 947 resulting in a change in the predicted amino acid sequence are listed in Table 1. The genes affected included those predicted to be involved in metabolism and energy production, transcriptional regulation, transporters, and putative virulence factors. Among the latter category, an SNP resulting in a L43P substitution was identified in *cpsE*, which encodes the glycosyl transferase that initiates assembly of the capsular polysaccharide (CPS) repeat unit. However, we have previously shown that there is no difference in total CPS production between 4559 and 947 (7). The SNP in the putative plasmin and fibronectin-binding protein gene *pfbA* is also a conservative T318M substitution. The protein encoded by *iga* is truncated in 4559 compared to 947, but only by four amino acids. On the other hand, the *nanB* sequence in 947 has a premature stop codon that truncates the protein by 330 amino acids (47% of the 4559 protein), presumably inactivating the gene product. Mutagenesis studies have previously shown that NanB contributes to colonization of both the upper and lower respiratory tract of mice, albeit to a lesser extent than the major neuraminidase NanA (8). Nevertheless, 4559 and 947 colonize the nasopharynx equally well (7). Interestingly, SNPs were identified in two metabolic genes, coding for ATP-dependent 6-phosphofructokinase (*pfkA*) and a glycogen synthase (*glgA*), as well as in two helix-turn-helix (HTH)-type transcriptional regulators, *scrR* and *rafR*, involved in metabolism of sucrose and raffinose, respectively. Given the importance of carbohydrate metabolism to S. pneumoniae (9), we employed a phenotypic microarray to compare the capacity of 4559 and 947 to metabolize over 100 different carbohydrates (see Materials and Methods). The only difference observed between the ear and blood isolates was a reduced capacity of the former (947) to grow in medium containing raffinose as the sole carbon source (data not shown).

**TABLE 1:** Genes containing indels or SNPs that led to amino acid changes, identified from the whole-genome variant calling analysis between 4559 and 947^*a*^

Locus tag in 947	Gene	Product	Change in aa sequence in 4559 relative to 947
0862	*pfkA*	ATP-dependent 6-phosphofructokinase	S212G
1153	*glgA*	Glycogen synthase	E174G
1345	*pncB*	Nicotinate phosphoribosyltransferase	N434D
1631	*scrR*	HTH-type transcriptional regulator	ΔL27-G28
**1803**	***rafR***	**HTH-type transcriptional regulator**	**D249G**
1255	*pyrP*	Uracil permease	V65A
1737	*piuA*	Fe^3+^ import ATP-binding protein	G141V
2020		ABC transporter ATP-binding protein	Y508N
0330	*cpsE*	CPS glycosyltransferase	L43P
1139	*iga*	Immunoglobulin A1 protease	Premature stop 1905 (4559) due to indel
1594	*nanB*	Sialidase B	Premature stop 362 (947) due to indel
1741	*pfbA*	Plasmin and fibronectin-binding protein A	T318M
0945	*coiA*	Competence protein	E78K
1141	*addA*	ATP-dependent helicase/nuclease subunit A	I980M
1060		Acetyl transferase	C101G
1194		Cytosolic protein containing multiple CBS domains	Premature stop 104 (947) due to SNP
1731		Hypothetical protein (no Pfam match)	H32P

a Results for the raffinose pathway gene *rafR* are in boldface.

Genetic differences between ear/blood isolate pairs that are common to two unrelated serotypes/ST lineages would be strong candidates for determinants of tissue tropism. Genomic comparisons were therefore also made between two serotype 3 ST180 ear and blood isolates (strains 180/2 and 180/15, respectively), which like the serotype 14 ST15 isolates, have previously been shown to exhibit distinct tissue tropism in mice in accordance with clinical isolation site (6). There were no differences in ARs between the two strains, while SNPs and indels impacting the deduced amino acid sequence for 27 genes were identified (Table 2). Interestingly, there were no affected genes in common with those in Table 1. However, an I227T SNP was detected in the serotype 3 *rafK* gene, encoding the ATP-binding protein component of the raffinose ABC transporter. RafK is known to be essential for activation of other *raf* operon genes, and the SNP identified in ST180 isolates is located in the conserved regulatory domain motif 1 (10). Thus, potential defects in raffinose uptake/metabolism appear to be a common feature of ear isolates from both serotypes/lineages.

**TABLE 2:** Genes containing indels or SNPs that led to amino acid changes, identified from the whole-genome variant calling analysis between 180/2 and 180/15^*a*^

Locus tag in 180/2	Gene	Product^*b*^	Change in aa sequence of 180/15 relative to 180/2
100	*purN*	Phosphoribosyl-glycinamide	G81A
254	*rpsJ*	30S ribosomal protein S10	Y58D
285		Hypothetical protein	A81S
314	*cdsA*	Phosphatidate cytidylyltransferase	M14I
335	*adhP*	Alcohol dehydrogenase 1	M210V
403	*fabK*	Enoyl-[acyl-carrier-protein] reductase	I1029T
449		Nitronate monooxygenase	I55M
512	*glnA*	Glutamine synthetase	F22L
645	*nhaK*	Sodium, potassium, lithium and rubidium/H^+^ antiporter	G190D
741		VanZ family protein	C149W
996		Hypothetical protein	H38R
1121	*clcA*	H^+^/Cl^−^ exchange transporter	M131I
1138	*ptsH*	Phosphocarrier protein HPr	I14V
1172		Formate/nitrate transporter	A211E
1194	*glnP*	Glutamine transport system permease protein	S662A
1234		SpF43_sRNA	Y31C
1306	*alaS*	Alanine-tRNA ligase	E18A
1387	*apbE*	FAD:protein FMN transferase	M52I
1404		LPXTG cell wall anchor domain-containing protein	ΔK112-Q119; G125K, E126T, P127E, E130V, K131N, I133D; ΔQ135-P178
**1491**	***rafK***	**Raffinose import ATP-binding protein**	**I227T**
1616	*dnaB*	DNA helicase	C375R
1760	*fepD_2*	Ferric enterobactin transport system permease protein	S248G
1863	*rpoC*	DNA-directed RNA polymerase subunit beta	D76E
1878	*acyP*	Acylphosphatase	V4I
1887	*rsgA*	Small ribosomal subunit biogenesis	G40S
2045	*aspS*	Aspartate tRNA ligase	E51V
2100	*dltD*	d-Alanyl-lipoteichoic acid biosynthesis protein	D151E

a Results for the raffinose pathway gene *rafK* are in boldface.

b FAD, flavin adenine dinucleotide; FMN, flavin mononucleotide.

### Blood isolates utilize raffinose more efficiently than ear isolates.

**FIG 1:**
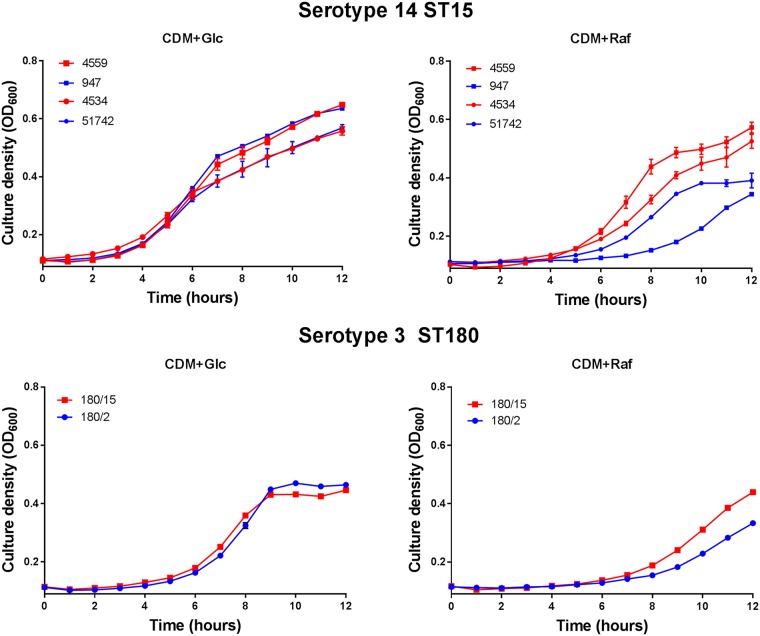
Differential growth of blood and ear isolates in raffinose. S. pneumoniae serotype 14 ST15 blood isolates 4559 and 4534 and ear isolates 947 and 51742 were grown in 200 µl CDM supplemented with 0.5% glucose (CDM+Glc) or 0.5% raffinose (CDM+Raf). Similar growth studies were also performed for serotype 3 ST180 strains 180/15 (blood isolate) and 180/2 (ear isolate). OD_600_ was measured every hour for 12 h. Data are mean OD_600_ ± standard deviation (SD) from triplicate assays.

In view of the SNPs in genes associated with raffinose metabolism between ear and blood isolates in two unrelated serotypes/STs and the fact that the serotype 14 ear and blood isolates differed only in their ability to metabolize raffinose on phenotypic microarray analysis, *in vitro* growth phenotypes were further investigated. Strains 4559 and 947, as well as another pair of serotype 14 ST15 blood and ear isolates (4534 and 51742, respectively), were grown in a chemically defined medium (CDM) with either glucose or raffinose as the sole carbon source (designated CDM+Glc and CDM+Raf, respectively) (Fig. 1). In CDM+Glc, there were no significant differences in growth rates between blood and ear isolates. However, in CDM+Raf, the two blood isolates grew at a higher rate and to a higher final culture density (optical density at 600 nm [OD_600_]) than either of the serotype 14 ST15 ear isolates. Similarly, there was no significant difference in growth rates of the serotype 3 ST180 ear and blood isolates (180/15 and 180/2, respectively) in CDM+Glc, but the blood isolate grew better than the ear isolate in CDM+Raf (Fig. 1). Thus, defective growth in raffinose appears to be a common defect in ear isolates relative to serotype/ST-matched blood isolates.

**FIG 2:**
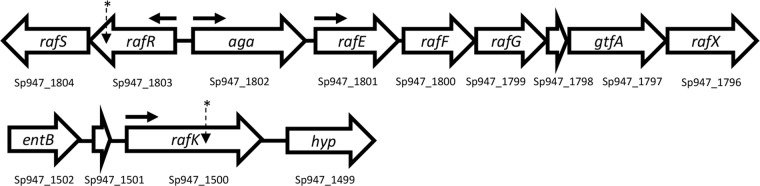
Genetic loci encoding raffinose uptake and utilization in S. pneumoniae. The numbers below each gene refer to the locus tags in the serotype 14 ST15 947 genome. The locations of SNPs in serotype 14 ST15 and serotype 3 ST180 isolates are indicated with asterisks; horizontal arrows show the locations of promoters.

**FIG 3:**
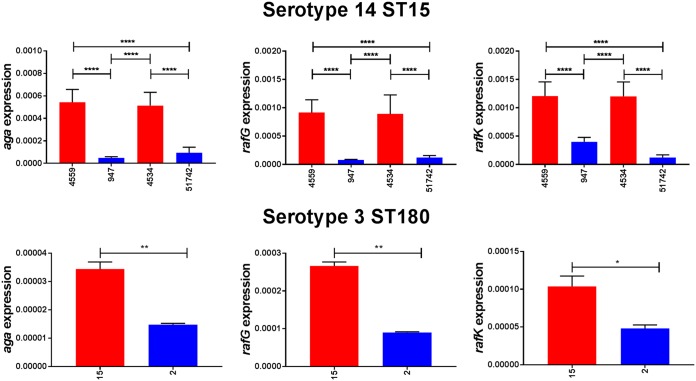
Expression of raffinose pathway genes by serotype 14 and 3 blood and ear isolates. The indicated strains were grown in CDM+Glc to an OD_600_ of 0.2, washed and resuspended in CDM+Raf, and then incubated at 37°C for a further 30 min. RNA was then extracted, and levels of *aga*, *rafG*, and *rafK* mRNA were analyzed by qRT-PCR using 16S rRNA as an internal control (see Materials and Methods). The data presented are the means ± SD from three independent experiments. *, *P < *0.05, **, *P < *0.01, and ****, *P < *0.0001, by unpaired *t* test.

The raffinose uptake/utilization operon in S. pneumoniae comprises genes encoding transcriptional regulators (*rafR* and *rafS*), an α-galactosidase (*aga*), the ABC transporter substrate-binding protein and two cognate permeases (*rafE*, *rafF*, and *rafG*), a sucrose phosphorylase (*gtfA*), and a protein of unknown function (*rafX*), as well as the ATP binding protein component of the transporter (*rafK*), which is independently located in the genome (11) (Fig. 2). To determine if the difference in ability to utilize raffinose between the blood and ear isolates corresponded with raffinose operon gene expression, S. pneumoniae serotype 14 ST15 strains 4559, 947, 4534, and 51742 and serotype 3 ST180 strains 180/2 and 180/15 were grown to the same OD_600_ (0.2) in CDM+Glc and then washed and resuspended in CDM+Raf and incubated for a further 30 min. RNA was then extracted, and levels of *aga*, *rafG*, and *rafK* mRNA, representative of each of the three *rafR*-regulated transcriptional units, were then measured relative to 16S rRNA by quantitative real-time reverse transcription-PCR (qRT-PCR). In every case, expression levels for all three genes were significantly greater in the blood isolates than in the respective ear isolates (Fig. 3).

**FIG 4:**
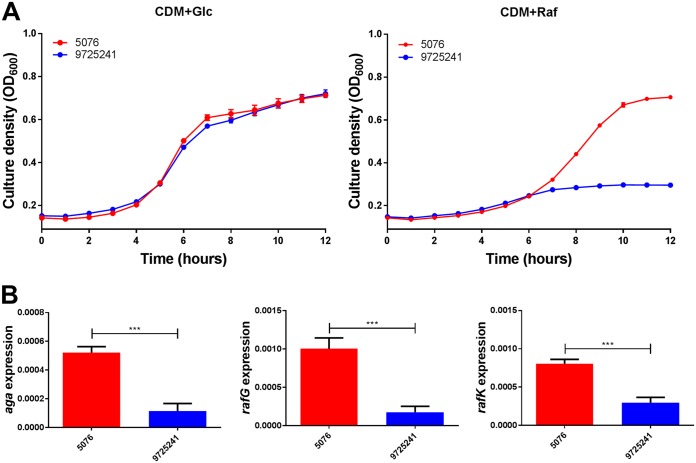
Growth phenotype and raffinose pathway gene expression in serotype 23F ST81 blood and ear isolates. (A) Growth of blood isolate 5076 and ear isolate 9725241 in CDM+Glc or CDM+Raf was monitored by OD_600_ for 12 h. Data are mean OD_600_ ± SD from triplicate assays. (B) The indicated strains were grown in CDM+Glc to an OD_600_ of 0.2, washed and resuspended in CDM+Raf, and then incubated at 37°C for a further 30 min. RNA was then extracted, and levels of *aga*, *rafG*, and *rafK* mRNA were analyzed by qRT-PCR using 16S rRNA as an internal control. The data presented are the means ± SD from three independent experiments. ***, *P < *0.001 by unpaired *t* test.

As further confirmation, blood and ear isolates belonging to serotype 23F ST81 were also tested for growth in CDM+Glc and CDM+Raf, as well as for expression of *aga*, *rafG*, and *rafK* (Fig. 4). Again, the blood isolate grew to a higher OD_600_ than the ear isolate in CDM+Raf, but not in CDM+Glc. Moreover, expression of all three *raf* genes was significantly higher in the blood isolate than in the ear isolate.

### The SNP in 947 *rafR* is responsible for its raffinose phenotype.

**FIG 5:**
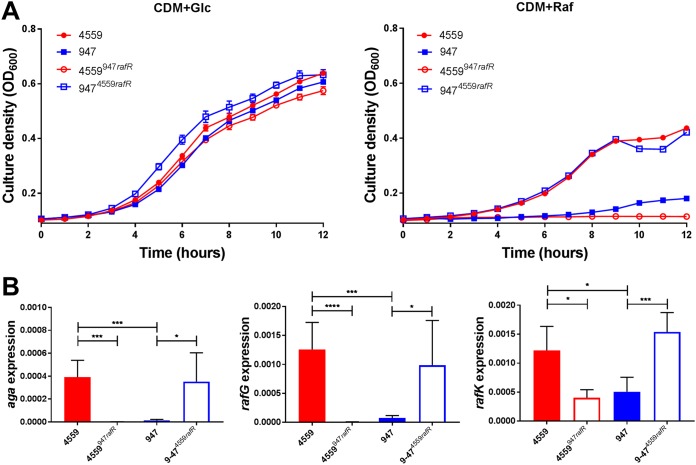
Growth phenotype and raffinose operon gene expression in *rafR* exchange mutants. (A) S. pneumoniae strains 4559, 947, 4559*^947rafR^*, and 947*^4559rafR^* were grown in CDM+Glc or CDM+Raf, and OD_600_ was monitored for 12 h. Data are mean OD_600_ ± SD from triplicate assays. (B) The indicated strains were grown in CDM+Glc to an OD_600_ of 0.2, washed and resuspended in CDM+Raf, and then incubated at 37°C for a further 30 min. RNA was then extracted, and levels of *aga*, *rafG*, and *rafK* mRNA were analyzed by qRT-PCR. Data are the means ± SD from three independent experiments. *, *P < *0.05, ***, *P < *0.001, and ****, *P < *0.0001, by unpaired *t* test.

In order to test whether the distinct *in vitro* and *in vivo* phenotype of 947 relative to 4559 was attributable to the SNP in *rafR*, allelic-exchange mutagenesis was performed in 4559 and 947, generating a 4559 derivative with its *rafR* allele replaced by that from 947 (designated 4559^947^*^rafR^*) and a 947 derivative expressing the 4559 *rafR* allele (947^4559^*^rafR^*) (see Materials and Methods). Growth assays in CDM+Glc showed no significant differences in growth rates between 4559, 947, 4559^947^*^rafR^*, and 947^4559^*^rafR^*. However, in CDM+Raf, growth of 4559^947^*^rafR^* was at least as poor as that of 947, while growth of 947^4559^*^rafR^* was similar to that of 4559 (Fig. 5A). Expression of *aga*, *rafG*, and *rafK* was then examined in 4559, 947, 4559^947^*^rafR^*, and 947^4559^*^rafR^* by qRT-PCR after 30 min of growth in CDM+Raf. For all three genes, expression levels in 947^4559^*^rafR^* were indistinguishable from those in 4559, while expression in 4559^947^*^rafR^* was essentially the same as that in 947 (Fig. 5B). Thus, exchange of *rafR* alleles between 4559 and 947 significantly impacts both growth phenotype and *raf* operon gene expression in CDM+Raf.

### Virulence phenotypes of 4559 and 947 and their *rafR* exchange mutants.

**FIG 6:**
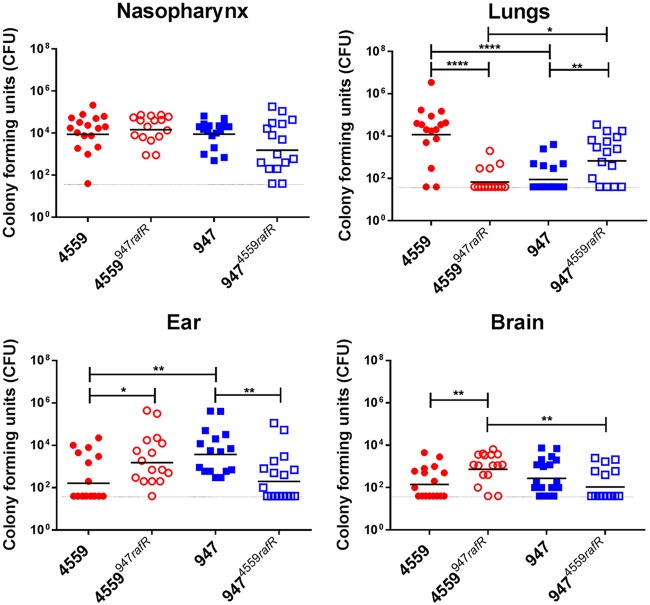
Virulence phenotype of *rafR* exchange mutants. Groups of 16 mice were infected intranasally with 10^8^ CFU of the indicated strain. At 24 h, all mice from each group were euthanized and numbers of pneumococci in the indicated tissues/sites were quantitated (see Materials and Methods). Viable counts (total CFU per tissue) are shown for each mouse at each site; horizontal bars indicate the geometric mean (GM) CFU for each group; the broken line indicates the threshold for detection. Differences in GM bacterial loads between groups are indicated by asterisks: *, *P < *0.05, **, *P < *0.01, and ****, *P < *0.0001, by unpaired *t* test.

In order to determine whether the marked difference in virulence phenotypes of 4559 and 947 is also directly attributable to the SNP in *rafR*, 4559, 947, 4559^947^*^rafR^*, and 947^4559^*^rafR^* were tested in a murine intranasal challenge model. Groups of Swiss mice were challenged with 10^8^ CFU of each strain, and bacterial loads were quantitated in various tissues 24 h postchallenge (Fig. 6). No significant differences in bacterial numbers in the nasopharynx were seen between any groups (Fig. 6), and no bacteria were detected in the blood of any mice (data not presented). However, 4559 was better able than 947 to persist in the lungs of infected mice, with significantly higher geometric mean (GM) bacterial load (*P < *0.0001) and a significantly greater proportion of infected animals (14/16 versus 6/16; *P < *0.01) (Fig. 6). On the other hand, bacterial loads of 947 in the ear were significantly greater than that for mice challenged with 4559 (*P < *0.01), and the proportion of infected mice was also significantly greater (16/16 versus 7/16; *P < *0.001) (Fig. 6). A similar trend was also seen in the brain (Fig. 6), in accordance with our previous report (4).

Exchanging the *rafR* alleles has a striking impact on virulence phenotype. In the lungs, both the GM CFU and proportion of infected mice for the group challenged with 4559^947^*^rafR^* were significantly lower than those for the 4559 group (*P < *0.0001 and *P < *0.001, respectively). Indeed, the virulence phenotype of 4559^947^*^rafR^* was indistinguishable from that of 947. Conversely, the GM bacterial load and proportion of infected mice for the 947^4559^*^rafR^* group were significantly greater than those for the 947 group (*P < *0.01 and *P < *0.05, respectively); there were no significant differences in these parameters between the 947^4559^*^rafR^* and 4559 groups. In the ear, both the GM CFU and proportion of infected mice for the group challenged with 4559^947^*^rafR^* were significantly greater than those for the 4559 group (*P < *0.05 and *P < *0.01, respectively). Conversely, both the GM CFU and proportion of infected mice for the group challenged with 947^4559^*^rafR^* were significantly lower than those for the 947 group (*P < *0.01 in both cases). Moreover, there was no significant difference in either GM bacterial loads or proportions of infected mice between the 4559^947^*^rafR^* and 947 groups or between the 947^4559^*^rafR^* and 4559 groups (Fig. 6). A similar pattern is seen in the brain; the GM CFU for the 4559^947^*^rafR^* group was significantly greater than those for either the 4559 or 947^4559^*^rafR^* groups (*P < *0.01 in both cases). Moreover, there was no significant difference in either GM bacterial loads or proportions of infected mice between the 4559^947^*^rafR^* and 947 groups or between the 947^4559^*^rafR^* and 4559 group (Fig. 6). Collectively, these data show that swapping the *rafR* allele between 4559 and 947 leads to a switch in their respective virulence profiles, and thus, the D49G SNP in *rafR* is entirely responsible for the observed difference in tissue tropisms between the serotype 14 ST15 blood and ear isolates.

### Mutagenesis of *rafK* in serotype 3 ST180 blood and ear isolates.

**FIG 7:**
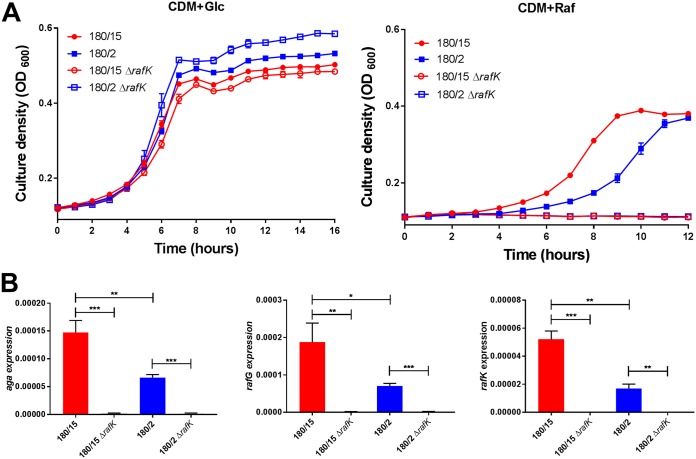
Growth phenotype and raffinose operon gene expression in Δ*rafK* mutants. (A) S. pneumoniae serotype 3 strains 180/15, 180/2, 180/15Δ*rafK*, and 180/2Δ*rafK* were grown in CDM+Glc or CDM+Raf, and OD_600_ was monitored for 12 h. Data are mean OD_600_ ± SD from triplicate assays. (B) The indicated strains were grown in CDM+Glc to an OD_600_ of 0.2, washed and resuspended in CDM+Raf, and then incubated at 37°C for a further 30 min. RNA was then extracted, and levels of *aga*, *rafG*, and *rafK* mRNA were analyzed by qRT-PCR. Data are the means ± SD from three independent experiments. *, *P < *0.05, **, *P < *0.01, and ***, *P < *0.001, by unpaired *t* test.

Attempts to construct *rafK* exchange mutants of serotype 3 ST180 blood and ear isolates (180/15 and 180/2, respectively) analogous to the *rafR* exchange mutants constructed for the serotype 14 strains were not successful. Thus, the impact of the SNP in RafK could not be directly tested. However, we were able to delete the native *rafK* genes from both type 3 strains (designated 180/15 Δ*rafK* and 180/2 Δ*rafK*, respectively). Both mutants were incapable of growth in CDM+Raf, and expression of *aga* and *rafG* was virtually undetectable by qRT-PCR; *rafK* expression was also undetectable, as expected (Fig. 7). Thus, the ear isolate 180/2 exhibits a phenotype that is intermediate between that of the blood isolate 180/15 and either of the two Δ*rafK* mutants, consistent with partial functionality of the ear isolate RafK. S. pneumoniae *rafK* deletion mutants have previously been shown to be outcompeted by the wild type in the murine lung and nasopharynx (10, 12). Similarly, in the present study, bacterial loads in the lungs, blood, ear, and brain were also lower for mice challenged with the 180/2 and 180/15 Δ*rafK* mutants relative to those challenged with the respective wild types at 48 h after intranasal challenge (result not presented). This indicates that even the intermediate level of raffinose pathway gene expression exhibited by ear isolate 180/2 contributes to virulence.

## DISCUSSION

Pneumococci are strictly fermentative bacteria, relying solely on carbohydrate metabolism for energy and growth (13). However, carbohydrate availability differs between host niches, and so the ability to respond to and utilize distinct carbohydrates is crucial for pneumococcal fitness *in vivo*. The S. pneumoniae genome encodes 21 phosphotransferase systems (PTSs) and up to 8 ATP binding cassette (ABC) transporters for the import of carbohydrates (9, 14), accounting for roughly 30% of all transport systems. Previous studies have shown that several of these carbohydrate transporters, present in both the core and accessory genome, impact pneumococcal virulence. For example, a sucrose PTS and ABC transporter system of serotype 4 pneumococci have been shown to play roles in murine colonization and pneumonia, respectively (15), while transporters for carbohydrates such as glucose, galactose, and mannose were shown to impact invasive pneumococcal disease (16–18).

The present study further underscores the critical role played by differential carbohydrate metabolism in pneumococcal pathogenesis. It demonstrates that reduced capacity to utilize raffinose does not simply reduce pneumococcal virulence, but rather changes the nature of disease caused. In multiple serotypes/ST lineages, ear isolates had defective growth in CDM+Raf and reduced expression of raffinose pathway genes relative to their serotype/ST-matched blood isolates. Exchange of *rafR* alleles between ear and blood isolates of serotype 14 ST15 reversed these *in vitro* phenotypes. Moreover, *rafR* exchange caused blood isolates to now cause otitis media and meningitis rather than pneumonia, after intranasal challenge, and conversely cause ear isolates to now target the lungs. This striking switch in *in vitro* and *in vivo* behaviors was attributable to a single, nonconservative SNP (D249G) in RafR, identifying this residue as one of critical functional importance. Significantly, the region of RafR from amino acids 226 to 268 comprises a conserved signature sequence for the AraC/XylS family of transcriptional regulators (11).

Interestingly, in spite of exhibiting similarly distinct *in vitro* and *in vivo* phenotypes, the serotype 3 ST180 blood and ear isolates did not share the SNP in *rafR*, but rather had an SNP in *rafK*, which encodes the ATPase required for raffinose uptake via the ABC transport system encoded by *rafEFG*. RafK-mediated uptake of raffinose has previously been shown to be essential for induction of the *raf* operons in S. pneumoniae D39 (10). Attempts to construct *rafK* exchange mutants in this lineage (analogous to the serotype 14 ST15 *rafR* exchange mutants) were not successful. However, *rafK* deletion mutants of both 180/2 and 180/15 were obtained. Whereas the wild-type ear isolate 180/2 exhibited reduced growth in CDM+Raf and expression of *aga*, *rafG*, and *rafK* relative to the wild-type blood isolate 180/15, both *rafK* deletion mutants were unable to grow in CDM+Raf at all, and expression of any of the *raf* operon transcripts was undetectable. Clearly, the RafK allele carried by 180/2 retains partial function. The I227T SNP that distinguishes the RafK alleles of 180/2 and 180/15 is located in the conserved regulatory domain motif 1. This domain is believed to be involved in the interaction between RafK and the enzyme dihydrolipoamide dehydrogenase (DLDH), which has been shown to modulate raffinose uptake and *raf* operon expression in S. pneumoniae D39 (10). In the murine model, both *rafK* deletion mutants exhibited reduced bacterial loads in multiple host niches relative to their respective wild-type strains, consistent with previous reports (10, 12).

Our findings provide an interesting example of convergent evolution, whereby pneumococci belonging to two unrelated serotypes/lineages exhibit SNPs in separate genes, each affecting raffinose uptake and utilization, which in turn correlate with distinct pathogenic profiles in both mice and humans (the latter by inference from the clinical isolation site). In S. pneumoniae D39, induction of expression of the *raf* operon gene *aga* required the presence of raffinose; reduced but nevertheless significant *aga* expression also occurred in a *rafR* knockout mutant (11). Thus, *raf* operon expression in pneumococci can be impacted either by defects in raffinose import (e.g., due to a defective RafK), such that insufficient exogenous raffinose (if present) is internalized to induce *raf* expression, or by functional defects in the transcriptional activator RafR, such that baseline levels of expression induced by the presence of raffinose are not further upregulated. The *raf* operons are part of the core genome of S. pneumoniae, and BLASTX analysis of available genomes shows that there is between 1% and 3% deduced amino acid sequence variation within any of the *raf* genes. Thus, SNPs are widespread, but it is not known which (if any) of these other SNPs impact the capacity to import or utilize raffinose or the virulence phenotype.

Notwithstanding the results presented above, the precise mechanism whereby differential raffinose uptake/utilization determines the virulence phenotype is uncertain. Raffinose is a plant-derived trisaccharide present in many staple foods, particularly beans and soy (19, 20). Although humans are unable to metabolize it, dietary raffinose is known to be absorbed by the intestinal epithelium (21), raising the possibility of at least small amounts being present on mucosal surfaces. As part of the present study, we confirmed that expression of *aga*, *rafG*, and *rafK* was not detectable by qRT-PCR when pneumococci are grown *in vitro* in media lacking raffinose. However, expression of all three genes was detected in RNA extracts of mouse lung tissue 6 h after intranasal challenge with either of the serotype 14 ST15 blood or ear isolates (results not shown). Since raffinose is the only known inducer of the *raf* operon in S. pneumoniae, this finding is strongly indicative of the presence of bioavailable raffinose in the murine lung. A potential complicating factor is that RafEFG is reported to be also capable of importing stachyose (14), while RafK has been reported to also energize uptake of sialic acid and maltotetraose via unrelated transporters (12). Thus, the SNPs observed in the present study could have pleiotropic effects. However, no differences in metabolism of these sugars between the serotype 14 ST15 blood and ear isolates were observed using phenotypic microarray analysis, and the serotype 3 ST180 strains were unable to grow in CDM with stachyose or sialic acid as the sole carbon source. Moreover, there was no significant difference in the growth rates of the ST180 blood and ear isolates when grown in CDM with maltotetraose (results not presented). A particularly intriguing finding of the present study was that lower raffinose uptake/utilization by the ear isolates provided an advantage over blood isolates in the ear compartment. Interestingly, exogenous raffinose has recently been shown to promote biofilm formation by Streptococcus mutans by promoting aggregation of extracellular DNA into the biofilm matrix. Biofilm formation was unaffected by deletion of the α-galactosidase gene *agaL*, indicating that the effect was unrelated to metabolism of any internalized raffinose (22). Thus, it is conceivable that the reduced capacity of S. pneumoniae ear isolates to assimilate (and thereby deplete) raffinose from the middle ear mucosa may similarly promote pneumococcal biofilm formation in that niche, leading to otitis media. Further studies are in progress in our laboratory to elucidate the precise molecular mechanism whereby fine-tuning of levels of expression of raffinose uptake and utilization genes can have such a profound impact on pathogenic profiles of clinical isolates of S. pneumoniae.

## MATERIALS AND METHODS

### Bacterial strains and growth conditions.

The S. pneumoniae strains used in this study are listed in Table 3. Cells were routinely grown in casein-based, semisynthetic liquid medium (C+Y) (23) or serum broth (SB) as required. Growth assays were performed using a chemically medium (CDM) comprising RPMI 1640 medium (Sigma), supplemented with amino acids, vitamins, choline, and catalase as described previously (24), with either 0.5% glucose or 0.5% raffinose. Bacteria were plated on Columbia agar supplemented with 5% (vol/vol) horse blood (BA) with or without gentamicin (40 µg/ml), kanamycin (500 µg/ml), or streptomycin (150 µg/ml) (as required) and incubated at 37°C in 5% CO_2_ overnight. For gene expression analyses, strains were grown in CDM+Glc medium to an OD_600_ of 0.2, before being incubated in CDM+Raf for 30 min.

**TABLE 3:** S. pneumoniae strains used in this study

Strain	Description	Source	Reference
4559	Serotype 14 ST15	Blood	7
947	Serotype 14 ST15	Ear	7
4534	Serotype 14 ST15	Blood	7
51742	Serotype 14 ST15	Ear	7
4559^947^*^rafR^*	4559 expressing 947 *rafR* gene		This study
947^4559^*^rafR^*	947 expressing 4559 *rafR* gene		This study
180/15	Serotype 3 ST180	Blood	6
180/2	Serotype 3 ST180	Ear	6
5076	Serotype 23F ST81	Blood	This study
9725241	Serotype 23F ST81	Ear	This study

### Genome sequencing.

S. pneumoniae strains were grown to mid-exponential phase in Todd-Hewitt broth supplemented with 1% yeast extract. Genomic DNA (gDNA) was extracted using the Qiagen genomic DNA buffer set with 100/g Genomic Tips according to the manufacturer’s instructions, except mutanolysin (20 U) and sodium deoxycholate (0.1%) were included to aid cell lysis. The gDNA was sequenced at the Ramaciotti Centre for Genomics (University of New South Wales, Sydney, Australia) on an Illumina MiSeq (250-bp paired-end reads), as well as a PacBio RSII instrument using one SMRT cell per strain, a 20-kb insert library, and the P6 polymerase and C4 sequencing chemistry.

### Bioinformatic analyses.

The Artemis Comparison Tool was used to compare genomes (25). MiSeq reads of 4559 and 947 and 180/15 and 180/2 were mapped to the assembled reference genome of the opposing strain with BOWTIE2 version 2.2.6 (26). Variant calling was then performed using SAMTools version 0.1.18 (27), and variants were mapped to coding sequences of the reference strain using BEDTools version 2.25.0 (28). Single nucleotide polymorphisms (SNPs) and insertions/deletions (indels) were filtered for those with scores of 100 or greater. Artemis was used to visualize SNPs and indels (29). Sanger sequencing was performed to confirm the SNPs in *rafR* and *rafK* (Australian Genome Research Facility, Adelaide).

### Phenotypic microarrays.

Carbon phenotype microarray analysis was performed on the serotype 14 ST 15 strains, using the PM microplates PM1 and PM2A (Biolog, Inc.), which tested for the catabolism of 190 different carbon sources. Each well of the microarrays contained a different carbon source. Briefly, cells were suspended in the provided buffer (as per the manufacturer’s instructions) to an *A*_590_ of 0.37. One hundred microliters of this suspension was added to the wells, and the *A*_590_ was measured after 17 h of incubation at 37°C. Catabolism was measured through the reduction of a colorless tetrazolium dye by NADH, produced during catabolic activity. Absorbance values above 0.65 after subtraction of that for the zero carbon source blank were considered positive.

### Growth assays.

Each tested strain was grown in CDM supplemented with either 0.5% Glc (CDM+Glc), 0.5% Raf (CDM+Raf), or no sugar (CDM) and then incubated at 37°C for 12 h in 96-well flat bottom plates (Costar). The OD_600_ was measured every 15 min using a SPECTRAmax M2 spectrophotometer (Millennium Science). All experiments were conducted in triplicate and repeated at least two times.

### qRT-PCR.

Differences in levels of gene expression were assayed by one-step relative quantitative real-time RT-PCR (qRT-PCR) in a Roche LC480 real-time cycler essentially as described previously (30). The specific primers used for the various genes are listed in Table 4 and were used at a final concentration of 200 nM per reaction. As an internal control, primers specific for 16S rRNA were employed. Amplification data were analyzed using the comparative critical threshold cycle (2^−ΔΔ^*^CT^*) method (31).

**TABLE 4:** Oligonucleotide primers used in this study

Primer	Sequence (5′→3′)	Reference
*rafR* Flank F	GCGAACGTAGGTTACAATCGT	This study
*rafR* R j tail	GGAAAGGGGCCCAGGTCTCTCTAGCATGTGCTACCTCCTACC	This study
*rafR* F j tail	CATTATCCATTAAAAATCAAAGGGGAAATCCTACCAAGCTGTCTACC	This study
*rafR* Flank R	CGAACGTAGTTCAGTGGTAGAA	This study
Janus F	CCGTTTGATTTTTAATGGATAATG	33
Janus R	AGAGACCTGGGCCCCTTTCC	33
*aga* F	AAGGTCAGAATGGTCCACAG	This study
*aga* R	GCTGGAAAATCAGCCATAAA	This study
*rafG* F	CCTATGGCAGCCTACTCCATC	This study
*rafG* R	GGGTCTGTGGAATCGCATAGG	This study
*rafK* F	AACGACGTAGCTCCAAAAGA	This study
*rafK* R	GCTGGTTTACGTTCCAAGAA	This study
16s rRNA F	GGTGAGTAACGCGTAGGTAA	34
16s rRNA R	ACGATCCGAAAACCTTCTTC	34
*rafR* sanger	AGTAGAAGAGCTGGTGTTTG	This study
*rafR* sanger	TCTGTGACTAAGCCAGTTTC	This study
*rafK* Flank F	AGGACTTGGTTCTTGTTGAG	This study
*rafK* R ery tail	TTGTTCATGTAATCACTCCTTCCTACCATGAGGTGAACTCC	This study
*rafK* F ery tail	CGGGAGGAAATAATTCTATGAGATCAGTTAATCTAGGGAGAG	This study
*rafK* Flank R	CTCAAAGGCAACTGGACAAC	This study

### Mutagenesis.

The *rafR* gene swap between serotype 14 ST15 4559 and 947 strains, to produce 4559^947^*^rafR^* and 947^4559^*^rafR^*, was achieved via allelic exchange mutagenesis utilizing the Janus cassette, as described previously (32, 33). This involved a three-step process in which endogenous *rpsL* (which confers streptomycin sensitivity) was first replaced with the streptomycin-resistant *rpsL1* allele by direct transformation of the blood and ear isolates. The Janus cassette (comprising a kanamycin resistance marker and a dominant counterselectable *rpsL*^+^ marker) was then used to replace the native *rafR* gene by direct transformation with a linear PCR product comprising the Janus cassette flanked by sequences 5′ and 3′ to *rafR* (selecting on kanamycin). In the final step, the Janus cassette in Kan^r^/Strep^s^ transformants is replaced by transformation with the alternative *rafR* allele and flanking sequences, counterselecting on streptomycin (loss of the Janus cassette reinstates the Strep^r^ phenotype). Gene swap constructs were confirmed by Sanger DNA sequencing (AGRF, Adelaide). The *rafK* gene was also deleted from serotype 3 ST180/2 and ST180/15 by direct transformation with a linear DNA fragment comprising an erythromycin resistance cassette flanked by sequences 5′ and 3′ to *rafK* generated by overlap PCR, essentially as previously described (34). The primers used are listed in Table 4. Mutant constructs were confirmed by PCR.

### Animal studies.

Animal experiments were approved by the University of Adelaide Animal Ethics Committee. Groups of outbred 6-week-old female Swiss (CD-1) mice were anesthetized by intraperitoneal injection of pentobarbital sodium (Nembutal; Rhone-Merieux) and challenged intranasally (i.n.) with 50 µl of bacterial suspension containing approximately 1 × 10^8^ CFU in SB (7). The challenge dose was confirmed retrospectively by serial dilution and plating on BA. Mice were euthanized by CO_2_ asphyxiation at 24 h, and then tissue samples (lungs, nasopharynx, brain, ear, and blood) were harvested and pneumococci enumerated in tissue homogenates as described previously via serial dilution and plating on plates containing BA plus gentamicin (35).

### Data availability.

Genome sequences have been deposited with ENA under accession no. SAMEA5092021, SAMEA5092022, SAMEA5092023, and SAMEA5092024, for strains 947, 4559, 180/2, and 180/15, respectively.
